# A comparison of health utility scores calculated using United Kingdom and Canadian preference weights in persons with alzheimer’s disease and their caregivers

**DOI:** 10.1186/s12955-016-0510-y

**Published:** 2016-07-18

**Authors:** Mingying Fang, Mark Oremus, Jean-Eric Tarride, Parminder Raina, Canadian Willingness-to-pay Study Group, Gerry Mugford, Marshall Godwin, Allen Huang, Yves Bacher, Juan-Manual Villalpando, Sudeep S. Gill, Krista L. Lanctôt, Nathan Herrmann, David Cowan, Robert Petrella, David B. Hogan, Philip E. Lee

**Affiliations:** School of Public Health and Health Systems, University of Waterloo, 200 University Avenue West, Waterloo, Ontario Canada; Department of Clinical Epidemiology and Biostatistics, McMaster University, 1280 Main Street West, Hamilton, Ontario Canada; Program for Assessment of Technologies in Health Research Institute, St. Josephs Healthcare Hamilton, 25 Main Street West, Hamilton, Ontario Canada

**Keywords:** Alzheimer’s disease, Caregiver, EQ-5D-3L, Health-related quality-of-life

## Abstract

**Background:**

The use of the EQ-5D to asses the economic benefits of health technologies has led to questions about the cross-population transferability of preference weights to calculate health utility scores. The aim of this study is to investigate whether the use of UK and Canadian preference weights will lead to the calculation of different health utility scores in a sample of persons with Alzheimer’s disease (AD) and their primary informal caregivers.

**Methods:**

We recruited 216 patient-caregiver dyads from nine geriatric and memory clinics across Canada. Participants used the EQ-5D-3L to rate their health-related quality-of-life (HRQoL). EQ-5D-3L responses were transformed into health utility scores using UK and Canadian preference weights. The levels of agreement between the two sets of scores were assessed using intraclass correlation coefficients (ICCs). Bland-Altman plots depicted individual-level differences between the two sets of scores. Differences in health utility scores were tested using the Wilcoxon signed rank sum test. A generalized linear model with a gamma distribution was used to examine whether participants’ socio-demographic characteristics were associated with their health utility scores.

**Results:**

The distributions of health utility scores derived from both the UK and Canadian preference weights were skewed to the left. The intraclass correlation coefficient was 0.94 (95 % CI: 0.92, 0.95) for persons with AD and 0.92 (95 % CI: 0.88, 0.94) for the caregivers. The Canadian weights yielded slightly higher median health utility scores than the UK weights for caregivers (median difference: 0.009; 95 % confidence interval: 0.007, 0.013). This finding persisted after stratifying by disease severity. Few socio-demographic characteristics were associated with the two sets of health utility scores.

**Conclusions:**

Health utility scores exhibited small and clinically unimportant differences when calculated with UK versus Canadian preference weights in persons with AD and their caregivers. The original UK and Canadian population samples used to obtain the preference weights valued health states similarly.

## Background

Alzheimer’s disease (AD) is a chronic neurodegerative condition that accounts for 60 to 70 % of all cases of dementia. Cognitive impairment, functional decline, and behavior and mood problems are the core features of AD [1]. AD and other dementias are the seventh leading cause of mortality and disability and the fourth leading cause of disease burden in high-income countries [2].

Health-related quality of life (HRQoL) is an individual’s dynamic perception of the impact of a health state upon physical, emotional, and cognitive function, social role performance, well-being, and life satisfaction [3]. HRQoL is an important means of assessing the impact of AD treatments because available therapies mitigate the symptoms of cognitive decline, but do not alter the progression of the disease [4].

The EQ-5D-3L is one of the most frequently used generic instruments to measure HRQoL [5–7]. Algorithms (preference weights) can be used to convert EQ-5D-3L responses into health utility scores (range: 0 [equivalent to death] to 1 [equivalent to full health]), which are employed in cost-utility analyses to calculate quality-adjusted life-years (QALYs). The original preference weights for the EQ-5D-3L were derived from the general UK population using the time trade-off (TTO) method [8]. Researchers generated a Canadian set of preference weights for the EQ-5D-3L using the TTO method and a sample of 1145 participants who belonged to a market research panel [9]. In the UK and Canadian studies, the researchers chose different sub-sets of health states from the 243 total possible health states on the EQ-5D-3L. These sub-sets were further divided into smaller groups for each participant to value using the TTO method. Regression analyses were employed to develop a set of beta coefficients that would serve as the preference weights to convert EQ-5D-3L responses into health utility scores.

This study investigated whether the use of UK and Canadian preference weights would lead to the computation of different health utility scores in a sample of persons with AD and their primary informal caregivers. The topic is important because studies based in populations without domestic sets of preference weights will often draw upon the preference weights of other populations, regardless of whether the other populations’ weights are transferable. Unless transferability is assessed, researchers cannot be certain whether another population’s weights will provide unbiased health utility scores in their population of interest.

This issue is important within the context of AD because health utility scores are essential components of cost-utility analyses. These analyses can influence reimbursement decisions for AD pharmacotherapies, as evidenced in 2006 when the results of a cost-utility analysis prompted the United Kingdom’s National Health Service to delist coverage of cholinetserase inhibitors for persons with mild AD. The impact of cost-utility analyses on treatment decision-making highlights the importance of ensuring the unbiased nature of the underlying health utility scores.

Recent work in Canada has echoed our sentiments about the use of adequate preference weights in cost-utility analyses [10]. Lien et al. point out that differences in country-specific preference weights could lead to differences in cost-utility results [10]. Similar concerns have also been raised in Italy [11]. In the context of Canada’s publicly-funded healthcare system, decisions regarding the efficient allocation of limited resources require support from unbiased analyses of cost-utility data. The issue extends beyond Canada to include any jurisidiction without a locally or domestically available set of preference weights. An examination of this issue may raise awareness among regulatory agencies that do not mandate the use of local or domestic preference weights.

To round out our objectives, we also explored whether socio-demographic factors might be associated with the health utility scores calculated in this study.

## Methods

### Subjects and data collection

Data were collected between November 2008 and August 2011 [12]. Two hundred sixteen persons with AD and their primary informal caregivers were recruited from nine memory or geriatric clinics across Canada. Eligible participants had a diagnosis of AD, as defined by the Diagnostic and Statistical Manual of Mental Disorders, Fourth Edition, Text revision criteria [13] or the National Institute of Neurological and Communicative Disorders and Stroke/Alzheimer’s Disease and Related Disorders Association criteria (NINCDS-ADRDA) [14]. We included persons with mild or moderate AD to ensure that participants would be cognitively capable of answering the study questions. The physicians who ran the recruiting clinics assessed disease severity using the Functional Assessment Staging in Alzheimer’s Disease Scale [15]. Participants’ primary informal caregivers also had to agree to participate in the study. All participants had to speak English or French.

We conducted separate one-on-one interviews with each participant. The interviews included socio-demographic questions (i.e., age, gender, education level, occupation and household annual income [Canadian dollars]) and the EQ-5D-3L. Each participant rated her or his own HRQoL. Caregivers did not provide proxy HRQoL ratings for persons with AD.

Prior to commencing each interview, participants read an information package about the study and they could ask the interviewer questions. The interviews began after participants signed an informed consent form. The study received ethics approval from the Hamilton Health Sciences/McMaster Health Sciences Research Ethics Board (project number 08-179) and from the local research ethics boards governing each of the nine recruitment sites.

We calculated health utility scores using the EQ-5D-3L responses and the UK [8] and Canadian preference weights [9]. We did not compute health utility scores for participants who failed to answer one or more of the EQ-5D-3L questions. Participants with missing health utility scores were excluded from statistical analyses involving these scores.

### Statistical analysis

Socio-demographic characteristics were summarized using medians and interquartile ranges for continuous variables, and frequencies for categorical variables. We used 1000 bootstrap samples to calculate bias corrected and adjusted 95 % confidence intervals (CIs) for all median health utility scores. We assessed the statistical significance of the median differences between the UK and Canadian health utility scores using the Wilcoxon signed rank sum test. Hodges-Lehmann’s methods for paired groups were employed to calculate the median differences and the 95 % confidence intervals for the median differences.

The overall agreement between the UK and Canadian health utility scores was assessed with the intraclass correlation coefficient (ICC), specifically the ICC(3,1) [16]. Bland-Altman plots [17] were created to graphically depict the difference in each participant’s health utility score (the score based on UK weights subtracted by the score based on Canadian weights).

We tested the association between socio-demographic factors (i.e., age, gender, education level, occupation, and annual household income) and each set of health utility scores using a generalized linear model (GLM) with a gamma distribution and bootstrap 95 % CIs for the regression coefficients. This type of GLM was shown to optimally fit the data compared to a generalized additive model, quantile regression by means of residual plots, and analysis of variance. We employed the Akaike Information Criterion (AIC) to choose the optimal model from among these different approaches. Based on the literature [18], we considered a change in health utility score of 0.074 to be a minimum clinically important difference (MCID).

Most analyses were carried out using R v3.2.0 (R Foundation for Statistical Computing, Vienna, Austria). To calculate the ICC (3,1), we used a two-way mixed-effects analysis of variance model and absolute agreement in SPSS v19 (IBM Corp., Armonk, NY).

## Results

A total of 216 persons with AD and their primary informal caregivers were included in the study (Table 1). The median age was 80 years for persons with AD and 69 years for caregivers. One-hundred five persons with AD (48.6 %) were female and the majority (*n* = 143, 66.2 %) of caregivers were female. Most persons with AD (*n* = 112, 51.9 %) did not exceed a high school education and all except one were retired. Most caregivers (*n* = 147, 68.1 %) had a post-secondary education and 140 (64.8 %) were retired. Most (*n* = 175, 81.0 %) persons with AD were diagnosed with mild AD and the rest were diagnosed with moderate AD. Over 50 % of participants in both groups reported no problems in all five EQ-5D-3 L dimensions (Table 2).

**Table 1:** Sample characteristics

Sample characteristics^a^		Persons with AD	Caregivers
		(*n* = 216)	(*n* = 216)
Age (years)		80 (78,81)	69 (59,77)
Gender	Female	105 (48.6)	143 (66.2)
Education^b^	High school or less	112 (51.9)	69 (31.9)
	College	35 (16.2)	50 (23.1)
	University	43 (20.0)	69 (31.9)
	Post-graduate	24 (11.1)	28 (13.0)
	Missing	2 (0.9)	0
Occupation	Retired	194 (89.8)	140 (64.8)
	Working	1 (0.5)	60 (27.8)
	Other^c^	21 (9.7)	16 (7.4)
Annual household income^d^	<$40,000	64 (29.6)	59 (27.3)
	$40,000-$80,000	42 (19.4)	78 (36.1)
	>$80,000	22 (10.2)	63 (29.2)
	No answer/Refused	88 (40.7)	16 (7.4)
Visual analogue scale (0 – 100)		80 (70,90)	80 (70,90)

Note. *AD* Alzheimer’s Disease

^a^Median (25^th^,75^th^ percentiles) for age and visual analogue scale; n (%) for all other variables

^b^College: some college or completed college; university: some univeristy or completed university; post-graduate: some university at Masters or Doctorate level or completed a Masters or Doctorate

^c^Homemaker, student, or unemployed

^d^Canadian dollars

**Table 2:** EQ-5D-3L responses

		Persons with AD – n (%)		Caregivers – n (%)	
		Age < 65 Years (*n* = 13)	Age ≥ 65 Years (*n* = 203)	Age < 65 Years (*n* = 76)	Age ≥ 65 Years (*n* = 140)
Mobility	No problems	12 (92)	140 (69)	69 (91)	81 (58)
	Some problems	1 (8)	62 (31)	7 (9)	59 (42)
	Confined to bed	0 (0)	1 (<1)	0 (0)	0 (0)
Self-care	No problems	12 (92)	181 (89)	76 (100)	134 (96)
	Some problems	1 (8)	21 (10)	0 (0)	6 (4)
	Unable to wash or dress	0 (0)	0 (0)	0 (0)	0 (0)
	Missing	0 (0)	1 (<1)	0 (0)	0 (0)
Usual activities	No problems	8 (62)	143 (70)	68 (90)	88 (63)
	Some problems	5 (38)	57 (28)	8 (10)	50 (36)
	Unable to wash or dress	0 (0)	2 (1)	0 (0)	2 (1)
	Missing	0 (0)	1 (< 1)	0 (0)	0 (0)
Pain/discomfort	No	12 (92)	133 (66)	51 (67)	60 (43)
	Moderate	1 (8)	69 (34)	24 (32)	76 (54)
	Extreme	0 (0)	1 (< 1)	1 (1)	4 (3)
Anxiety/depression	No	8 (62)	151 (74)	52 (68)	92 (66)
	Moderate	5 (13)	48 (24)	23 (30)	46 (32)
	Extreme	0 (0)	3 (2)	1 (1)	2 (1)
	Missing	0 (0)	1 (< 1)	0 (0)	0 (0)

Note. *AD* Alzheimer’s Disease

**Fig. 1:**
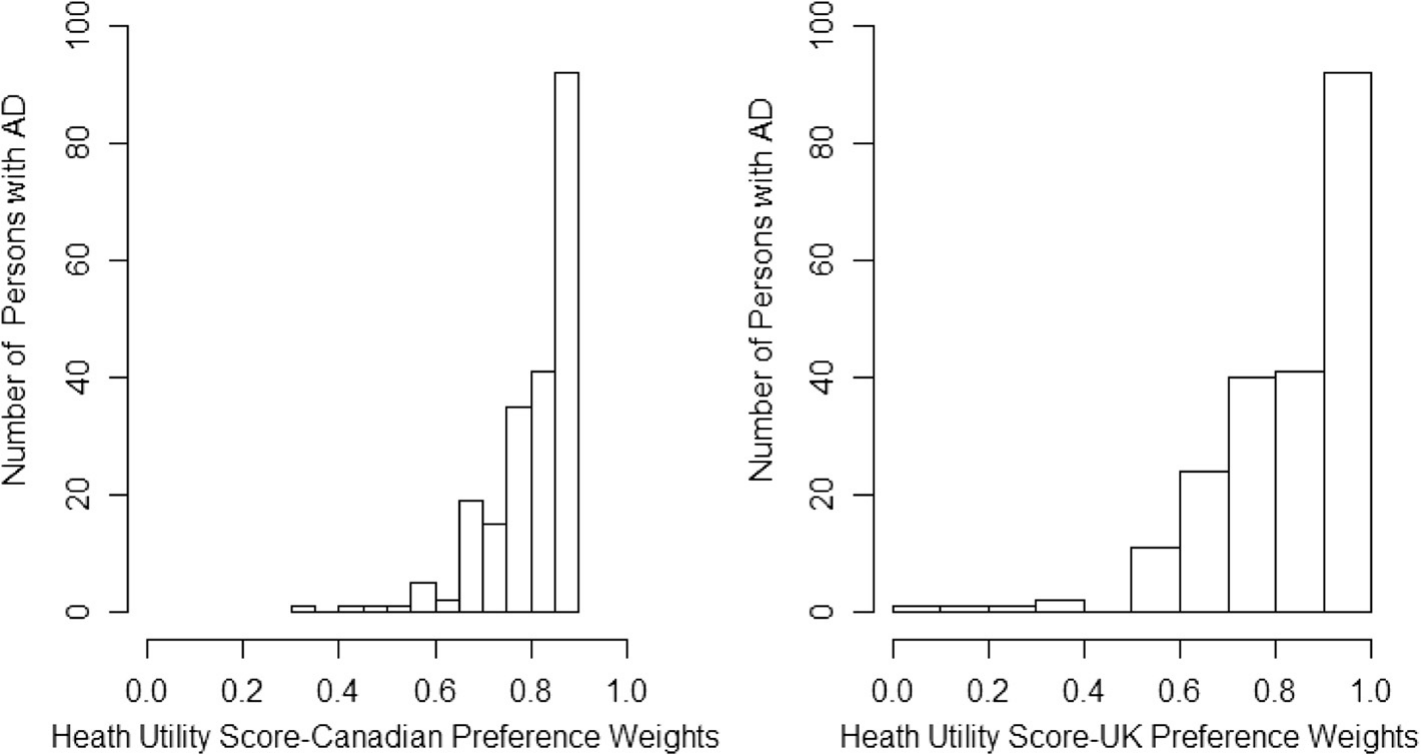
Distribution of health utility scores for persons with Alzheimer’s disease. AD = Alzheimer’s disease; UK = United Kingdom

**Fig. 2:**
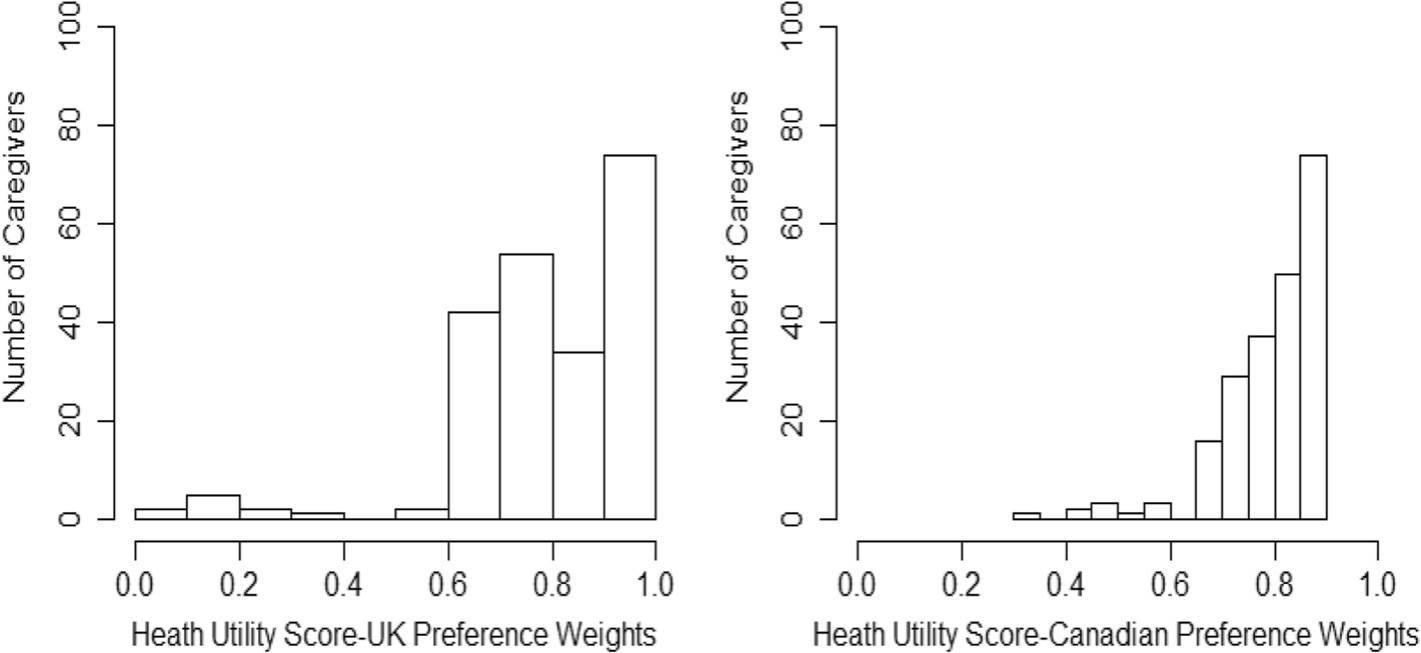
Distribution of health utility scores for caregivers. UK = United Kingdom

For persons with AD and caregivers, the distributions of health utility scores derived from the UK and Canadian preference weights were left-skewed (Figs. 1 and 2). Half of the persons with AD had health utility scores above 0.85 (UK weights) or 0.84 (Canadian weights). Similarly, half of the caregivers had scores above 0.80 (UK weights) or 0.83 (Canadian weights). We could not compute health utility scores for three persons with AD because they did not answer all of the questions on the EQ-5D-3L.

The difference between the two sets of health utility scores was not statistically significant in persons with AD (*p* = 0.63) (Table 3). However, the difference was statistically significant in the caregivers (median difference: 0.009 [95 % CI: 0.007, 0.013]), with the median score being higher (higher meaning better health) when calculated with the Canadian versus UK preference weights. Within each set of preference weights (UK versus UK, Canadian versus Canadian), the difference in health utility scores between persons with AD and caregivers was not statistically significant.

**Table 3:** Median EQ-5D-3L health utility scores

	UK-based preference weights (95 % CI)	Canadian-based preference weights (95 % CI)
Persons with AD	0.85 (0.81, 0.88)^a,b^	0.84 (0.83, 0.84)^a,c^
Caregivers	0.80 (0.80, 0.85) ^b,d^	0.83 (0.83, 0.83) ^c,d^

Note. *AD* Alzheimer’s disease, *CI* confidence interval, *UK* United Kingdom, Health utility scores were not calculated for three persons with AD who did not answer all of the EQ-5D-3L questions

^a^ *p* = 0.63 for median difference in persons with AD (UK versus Canadian weights)

^b^ *p* = 0.06 for median difference in persons with AD versus caregivers (UK weights)

^c^ *p* = 0.35 for median difference in persons with AD versus caregivers (Canadian weights)

^d^ *p* < 0.0001 for median difference in caregivers (UK versus Canadian weights)

The Canadian utilities were higher than the UK utilities in the caregiver group when the caregivers were stratified according to the disease severity (mild, moderate) of the persons under their care (Table 4). Median differences were 0.009 (95 % CI: 0.007, 0.013) in the mild subgroup and 0.013 (95 % CI: 0.007, 0.028) in the moderate subgroup. For persons with AD, the UK and Canadian health utility scores did not differ significantly.

**Table 4:** Median EQ-5D-3L health utility scores stratified by disease severity

	Severity	UK-based preference weights (95 % CI)	Canadian-based preference weights (95 % CI)
Persons with AD	Mild (*n* = 173)	0.85 (0.85,0.88)^a^	0.84 (0.84,0.84)^a^
	Moderate (*n* = 40)	0.81 (0.73,0.88)^b^	0.82 (0.74,0.84)^b^
Caregivers	Mild (*n* = 175)	0.81 (0.80,0.85)^c^	0.83 (0.83,0.83)^c^
	Moderate (*n* = 41)	0.80 (0.80,0.80)^d^	0.83 (0.80,0.84)^d^

Note. *AD* Alzheimer’s disease, *CI* confidence interval, *UK* United Kingdom, Health utility scores were not calculated for three persons with AD who did not answer all of the EQ-5D-3L questions

^a^ *p* = 0.59 for median difference in persons with AD within mild stratum

^b^ *p* = 0.98 for median difference in persons with AD within moderate stratum

^c^ *p* < 0.0001 for median difference in caregivers within mild stratum

^d^ *p* = 0.009 for median difference in caregivers within moderate stratum

**Fig. 3:**
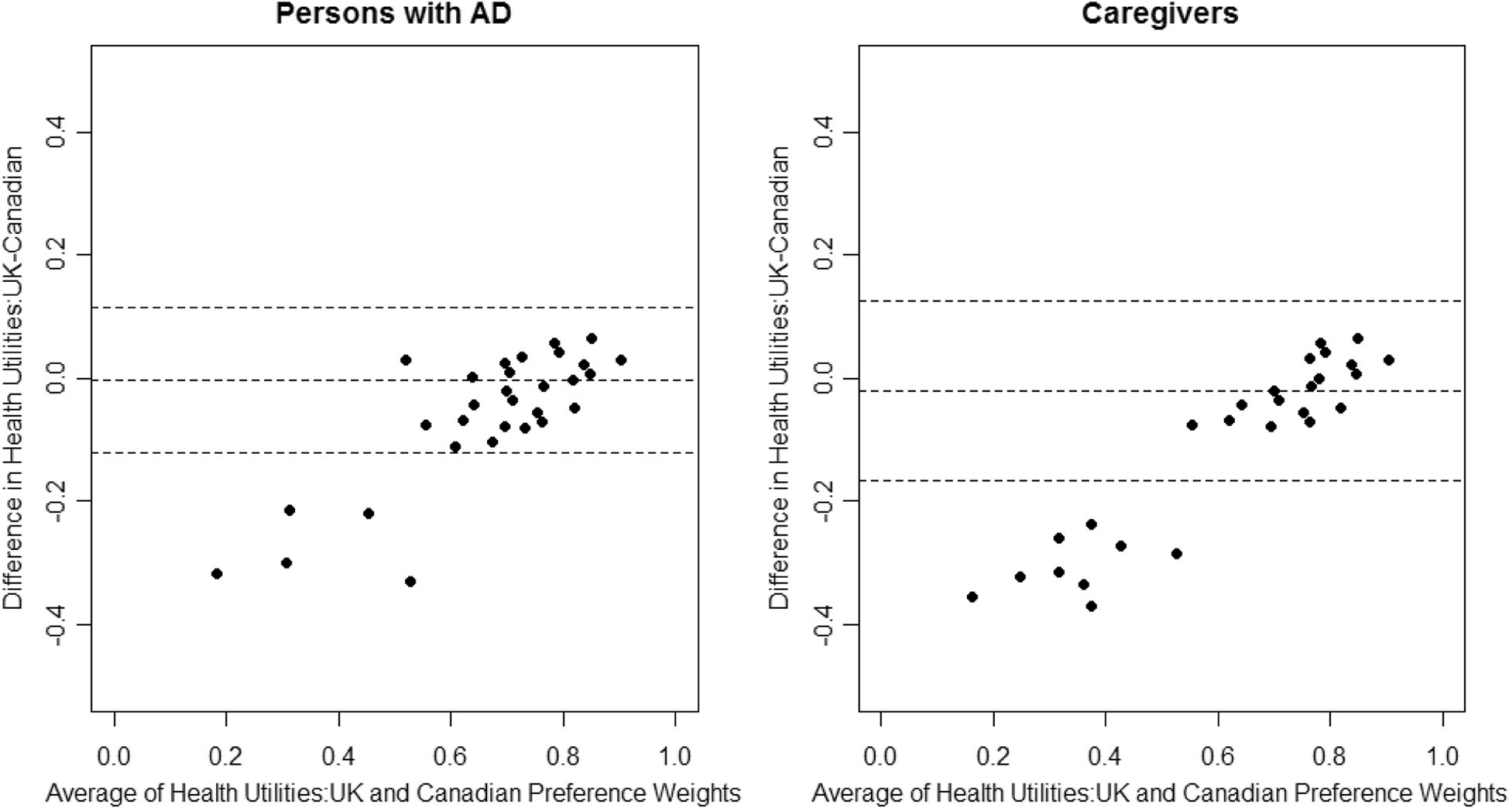
Bland-Altman plots. AD = Alzheimer’s disease; UK = United Kingdom

The overall agreement between health utility scores using UK and Canadian preference weights was high. The ICC (3,1)s were 0.94 (95 % CI: 0.92, 0.95) for persons with AD and 0.92 (95 % CI: 0.88, 0.94) for caregivers. According to the Bland-Altman plots, 95 % of the >individual differences in health utility scores (UK – Canadian) fell within a range of -0.12 to 0.12 for persons with AD and within -0.16 to 0.12 for caregivers (Fig. 3). For persons with AD, only 15 (7 %) of the individual differences in score exceeded the MCID of 0.074; for caregivers, the number was also 15 (9 %).

With respect to socio-demographic characteristics, only some education categories were associated with the two sets of health utility scores (Table 5). The scores for persons with AD who had a post-graduate education were an average of 0.1 points lower (UK weights) or 0.059 points lower (Canadian weights) than the scores for persons who had a high school or less education. The scores for caregivers with a post-graduate education were an average of 0.116 points lower (UK weights) or 0.04 points lower (Canadian weights) than the scores for caregivers with a high school or less education. Caregivers with a university education also had average scores that were 0.131 points lower than caregivers with a high school or less education. No other socio-demographic variables were statistically significantly associated with either set of health utility scores. Three of the statistically significant associations exceeded the MCID of 0.074.

**Table 5:** Multivariable relationship between socio-demographic variables and health utility scores

	UK-based preference weights		Canadian-based preference weights	
	Persons with AD	Caregivers	Persons with AD	Caregivers
	Regression coefficient (95 % CI)	Regression coefficient (95 % CI)	Regression coefficient (95 % CI)	Regression coefficient (95 % CI)
Age (years)	−0.004 (-0.053, 0.033)	−0.011 (-0.092, 0.055)	−0.002 (-0.011, 0.002)	0.008 (-0.051, 0.034)
Gender				
Female	Ref	Ref	Ref	Ref
Male	−0.039 (-0.110, 0.019)	−0.060 (-0.211, 0.067)	−0.022 (-0.072, 0.051)	0.036 (-0.119, 0.110)
Education^a^				
High school or less	Ref	Ref	Ref	Ref
College	−0.028 (-0.094, 0.127)	−0.105 (-0.237, 0.022)	−0.015 (-0.065, 0.070)	−0.047 (-0.134, 0.015)
University	−0.022 (-0.133, 0.016)	−0.131 (-0.253, -0.009)	−0.012 (-0.079, 0.073)	−0.074 (-0.142, 0.008)
Post-graduate	−0.100 (-0.181, -0.018)	−0.116 (-0.152, -0.006)	−0.059 (-0.080, -0.007)	−0.040 (-0.127, -0.009)
Occupation^b^				
Retired	Ref	Ref	Ref	Ref
Employed	0.046 (-0.075, 0.057)	−0.047 (-0.134, 0.059)	0.025 (-0.171, 0.102)	−0.034 (-0.078, 0.049)
Other	0.035 (-0.067, 0.063)	−0.015 (-0.117, 0.063)	0.017 (-0.024, 0.057)	−0.014 (-0.007, 0.037)
Household income^c^				
<$40000	Ref	Ref	Ref	Ref
≥$40000 to < $80000	0.004 (-0.087, 0.019)	0.028 (-0.131, 0.045)	0.003 (-0.035, 0.093)	−0.020 (-0.088, 0.017)
≥$80000	−0.015 (-0.039, 0.000)	0.002 (-0.006, 0.008)	−0.007 (-0.011, 0.021)	−0.064 (-0.141, 0.010)

Note. *AD* Alzheimer’s disease, *CI* confidence interval, *Ref* reference category, *UK* United Kingdom

^a^College: some college or completed college; university: some univeristy or completed university; post-graduate: some university at Masters or Doctorate level or completed a Masters or Doctorate

^b^Homemaker, student, or unemployed

^c^Canadian dollars

## Discussion

In this study of persons with AD and their primary informal caregivers, health utility scores derived from UK and Canadian preference weights exhibited slight differences from one another. Based on the MCID, these differences were not large enough to be considered clinically important. We could not find other studies that compared health utility scores calculated with UK and Canadian preference weights.

Evidence suggests health utility scores can be similar for people across countries with comparable socio-demographic characteristics (i.e., UK, Holland, and Germany [19] Spain and Germany [20]). Given the socio-demographic parallels between the UK and Canadian populations, the samples used to derive the UK and Canadian preference weights [8, 9] may have simply valued their health states similarly to one another. Thus, the health utility scores derived from each set of preference weights in this study did not differ appreciably from one another.

On the other hand, not every population with similar socio-demographic characteristics will place comparable value on the same health states. Representative samples of the United States (US) and UK populations valued 42 EQ-5D-3L health states and the adjusted mean difference in health utility scores was 0.10 points higher in the US population [21]. An earlier study involving the same sample as in the present study compared health utilities calculated with US and Canadian preference weights [12]. This comparison showed that Americans and Canadians agreed on the types of health states that should be considered ‘good’ or ‘bad’, but Canadians tended to place a lower value than Americans on most of these health states. When calculated with Canadian versus American preference weights, the mean health utility score was 0.06 points lower (95 % CI: -0.07, -0.06) in persons with AD and 0.05 points lower (95 % CI: -0.06, -0.04) in caregivers.

The findings reported in the literature raise a caution for researchers who wish to calculate health utility scores for study samples drawn from populations for which preference weights do not exist. The default practice in these situations has been to use preference weights derived from similar populations, but this approach could lead to over- or under-estimates of health utility scores. In the absence of preference weights for the population of interest, one can never be certain whether another population’s weights will provide unbiased results.

The caution about transferability of weights also applies to the EQ-5D-5L, which measures the same five health dimensions as the EQ-5D-3L, but expands the number of response options from three to five [22]. Recent work involving the EQ-5D-5L suggests that one set of preference weights may not capture inter-regional differences in a single country’s population if the population is spread over a large geographic area [23].

In our study, only a small number of socio-demographic characteristics affected the UK and Canadian health utility scores. Perhaps disease-specific factors, rather than socio-demographic characteristics, could better explain the scores in AD samples. In persons with AD, some studies have shown that health utility scores are affected by levels of depression and functional ability [24, 25]. Other work has suggested that a more complex series of socio-demographic and disease factors combine to modify the relation between disease severity and health utility scores [26]. For caregivers, health utility scores may be influenced by the extent to which patients are dependent on care, the perceived burden of being a caregiver, and the time involved in providing care [27, 28]. Researchers should consider these additional variables when they design studies to explain health utility scores in AD samples.

Our study found that most caregivers’ health utility scores were lower than the scores of persons with AD. The burden of caring for a person with AD may adversely affect a caregivers’ HRQoL [29]. Meanwhile, the effects of cognitive impairment might prevent persons with AD from perceiving the full impact of disease on their lives, therefore leading to high ratings of HRQoL [30, 31].

Few studies could be found that assessed the HRQoL of AD caregivers. One study conducted on the Canary Islands reported that the frequency of having at least some problems on each EQ-5D-3L dimension was greater in 237 AD caregivers compared to the islands’ general population [27]. However, the authors did not convert EQ-5D-3L responses into health utility scores. The caregiver utility scores in our study were somewhat lower than the scores reported in two studies (i.e., 0.87 [32], 0.88 [33]) of general adult populations in Canada, and about the same as the scores reported in a third Canadian study (i.e., 0.85 female, 0.81 male) [34].

Readers should take certain issues into account when interpreting the results of this study. The participants were recruited from geriatric or memory clinics, so they are unlikely to be representative of the average person with AD or the average caregiver. The participants with AD may be a healthier subset of all patients and the caregivers may be more informed about AD.

## Conclusion

Health utility scores exhibited some small yet clinically unimportant differences when calculated with UK versus Canadian preference weights in a sample of persons with AD and their caregivers. The UK and Canadian populations used to obtain the preference weights valued health states similarly to one another.

## Abbreviations

AD, Alzheimer’s disease; AIC, Akaike Information Criterion; CI, confidence interval; GLM, generalized linear model; HRQoL, health-related quality-of-life; ICC, intraclass correlation coefficient; MCID, minimum clinically important difference; NINCDS-ADRDA, National Institute of Neurological and Communicative Disorders and Stroke/Alzheimer’s Disease and Related Disorders Association; QALY, quality-adjusted life-year; TTO, time trade-off; UK, United Kingdom
